# Do hormonal contraceptives stimulate growth of neurofibromas? A survey on 59 NF1 patients

**DOI:** 10.1186/1471-2407-5-16

**Published:** 2005-02-09

**Authors:** Marge Lammert, Victor-Felix Mautner, Lan Kluwe

**Affiliations:** 1 Department of Maxillofacial Surgery, University Hospital Eppendorf, Hamburg, Germany

## Abstract

**Background:**

Neurofibromas are benign tumors of the peripheral nerves and hallmark of neurofibromatosis type 1 (NF1), a tumor suppressor gene syndrome. Neurofibromas mostly start developing at puberty and can increase in size and number during pregnancy. Expression of progesterone receptors has been found in 75% of the tumors. Many female NF1 patients are thus concerned about the possibility that hormonal contraceptives may stimulate the growth of their neurofibromas.

**Methods:**

A survey was carried out on 59 female NF1 patients who are practicing or have practiced hormonal contraception to examine the effect of the various contraceptives on the growth of neurofibromas.

**Results:**

Majority (53 out of 58) of patients who received oral estrogen-progestogen or pure progestogen preparations reported no associated tumor growth. In contrast, significant tumor growth was reported by two patients who received depot contraceptive containing high dose of synthetic progesterone.

**Conclusions:**

Oral contraceptives do not seem to stimulate the growth of neurofibromas in NF1 patients. High doses of progesterone might stimulate the growth of neurofibromas and deserve more caution.

## Background

Neurofibromatosis type 1 (NF1) is a genetic disorder with an incidence of about 1 in 3000. Multiple neurofibromas are the most significant hallmark of NF1. These benign tumors of peripheral nerves mostly start developing at puberty and can increase in size and number during pregnancy [1]. Since decades, physicians involved in the care of NF1 patients are concerned by the dilemma if hormonal contraceptives containing estrogen and progestogen could stimulate the onset of new or the growth of the yet present neurofibromas. Recently, McLaughlin & Jacks [2] reported expression of progesterone receptors and estrogen receptors in 75% and 5% of 59 neurofibromas immunohistochemically, respectively. The authors thus inferred an important role of progesterone in neurofibroma growth and suggested that antiprogestins may be useful in the treatment of this tumor.

Hormonal contraceptives contain synthetic progestogen which bind to the progesterone receptors. Depending on the formulation of the currently available preparation, combined oral contraceptives contain 0.02 to 0.05 mg synthetic estrogen and low doses of various types of synthetic progestogen. These kinds of contraceptives suppress the pituitary gonadotropin secretion and thus reduce the endogenous levels of estrogen and progesterone. The deficiency of endogenous estradiol is balanced by the exogenous supply of ethinylestradiol. Synthetic progestogen bind to progesterone receptors and thus compensate the deficiency of endogenous progesterone to certain extent. Progestogen-only preparations, such as the so-called progestogen-baby-pills, contain progestogen that is below the ovulation-inhibiting dose. This kind of pills only suppress the peak levels of the gonadotropins and thus reduce the estrogen and progesterone level to certain degree. In contrast, parenteral progestogen preparations (depot contraceptives) contain high doses of medroxyprogesterone acetate (150 mg) or norethisteron enanthat (200 mg). In the first days after the administration of these preparations, the blood concentration of progestogen is very high which decreases slowly over weeks.

To examine the effect of hormonal contraceptives on the behaviour of neurofibromas, we carried out a survey on 59 female NF1 patients in this study.

## Methods

NF1 was diagnosed according to the NIH criteria [3]. The protocol was approved by the institutional review board and all participants provided informed consent. A total of 110 female NF1 patients of the NF-Clinic Hamburg, Germany, were asked to fill out a question form (appendix in additional file 1) and some of them were interviewed personally by the authors. Only patients who have or had neurofibromas were included in this survey. The age of the included patients was between 18 and 80 years. Data collection was done from Aug. to Dec. 2003, except for one patient who changed preparation in Dec. 2003 and provided us new information recently. The major information acquired were: age of menarche, paramenia, contraceptive means and behaviour of their neurofibromas (appendix in additional file 1). Patients were asked to describe the increase of the growth of their neurofibromas as either slight, medium, significant or no (appendix in additional file 1).

## Results

Among the total of 110 patients included in the survey, 69 were practicing or had practiced hormonal contraception. Sixty-three received oral estrogen-progestogen preparations, 3 had pure progestogen and one had been given a parenteral depot contraceptive containing very high dose of medroxyprogesterone acetate (150 mg) and norethisterone enanthate (200 mg). For two patients, the names of the preparations could no longer be recalled. Eight out of the 63 patients who used oral estrogen-progestogen could not recall whether there was any change in the behaviour of their neurofibromas in association with hormonal contraception and were excluded from further evaluation.

Data from a total of 59 patients were thus available for evaluation (table 1). The period of hormonal contraception was between 3 months and 22 years among these 59 patients. Fifty three (91%) out of the 58 patients who used combined estrogen-progestogen preparation or pure progestogens were convinced that there was no tumor growth in association with the practiced hormonal contraception. Other five patients reported a slight increase in the size or/and number of their neurofibromas in the first few months of the hormonal contraception. These 5 patients used combined estrogen-progestogen preparation, containing 0.03 to 0.05 mg of estrogen and 0.125 to 2.5 mg synthetic progestogen (table 2). However, no tumor growth was reported by other 15 patients who also used the same preparation (table 2).

One patient received a depot progestogen (Depot-Clinovir) and reported a strong growth of neurofibromas and intraspinal tumors right after begin of the hormonal contraception. In December 2003, after the closure of our data collection, one patient changed from a combined contraceptive to the depot contraceptive Depot-Clinovir. Recently, she reported experience of rapid growth of her neurofibromas since the change. Interestingly, her tumors had been stable during the two years she took combined contraceptive (Ministon, Leios).

Of the 7 patients whose neurofibromas had increased in size and number upon hormonal contraception, two had been treated for paramenia as adolescents, while the other 4 were gynecologically normal.

## Discussion

Our results suggest that in majority of cases, combined hormonal contraceptives containing estogen and progestogen do not seem to stimulate growth of neurofibromas in NF1 patients. The reported slight tumor growth in 5 cases may not necessarily be the consequence of the contraceptives since other patients who used the same preparation did not notice related tumor growth (table 2). Our finding seems to eliminate the previous uncertainty and excessive caution in using hormonal contraceptives which often mean exporing NF1 patients to more severe problems as undesired pregnancies with important effects on tumoral growth.

The significant tumor growth associated with depot contraceptive in two cases suggests that high doses of medroxyprogesterone acetate and nerethisterone enanthate might stimulate the growth of neurofibromas in some cases. However, only two cases are far too few for further speculation. Additional reports and reviews regarding response of NF1 patients to this form of contraception will be very helpful.

Our survey was done *a posteriori *and mostly by a questionnaire. The results thus report only subjective impressions of patients, not an objective and quantifiable judgment of the researcher. This is clearly a major limitation of the study. Use of cultured Schwann cells and fibroblasts from human neurofibromas as well as recently developed mouse models of neurofibromas may help to further dissect the role of progesterone in regulating neurofibroma growth.

## Conclusions

Oral contraceptives do not seem to stimulate the growth of neurofibromas in most cases and thus may be used by NF1 patients. High doses of progesterone might stimulate the growth of neurofibromas and deserve closer observation.

## Competing interests

The author(s) declare that they have no competing interests

## Authors' contributions

ML has carried out the survey and prepared the preliminary version of the manuscript.

LK has prepared and completed the manuscript. She is corresponding author.

VM was responsible for the dianogis of the NF1 patients.

All authors read and approved the final manuscript.

## Pre-publication history

The pre-publication history for this paper can be accessed here:



## Supplementary Material

Additional File 1 Questionary for patients Click here for file

## Figures and Tables

**Table 1:** A survey on 59 female NF1 patients practicing hormonal contraception

	No alteration in tumors behavior	Slightly increase in tumor size or/ and number	Significant tumor growth	Total
Estrogen/progestogen, oral	50^a^	5		55^a^
Pure progestogen, oral	3	0		3
Pure progestogen, depot			2^a^	2^a^

^a^One patient had received oral estrogen/progestogen until Dec. 2003, and then changed to depot pure progestogen.

**Table 2:** Contraceptives reported to be associated with tumor growth

Contraceptive (Commercial name)	Type	Composition	Number of patients who experienced tumor growth	Number of patients who did not experience tumor growth
Non-Ovlon	combined oral	0.05 mg estrogen 1.0 mg noreethisteron acetate	1 slight	7
Marvelon	combined oral	0.03 mg estrogen 0.15 mg desogestrel	1 slight	6
Femranette	combined oral	0.03 mg estrogen 0.15 mg levonorgestrel	1 slight	1
Trisiston	combined oral	0.03 mg estrogen 0.125 mg levonorgestrel	1 slight	1
Ovanon	combined oral	0.05 mg estrogen 2.5 mg lynestrenol	1 slight	1
Depot-Clinovir	parenteral progestogen	150 mg medroxyprogesterone acetat 200 mg norethisteron enanthat	2 significant	0
