# Supplementary material for: Do hormonal contraceptives stimulate growth of neurofibromas? A survey on 59 NF1 patients
Source: BMC Cancer. 2005 Feb 9;5:16. doi: 10.1186/1471-2407-5-16 (PMC549555; doi:10.1186/1471-2407-5-16)
Supplement: Additional File 1 — Questionary for patients [file 1471-2407-5-16-S1.doc]

**Appendix: Questionary for patients**

Family name: First Name: Birth date:

Adress: Phone No.:

(1) normal menstruation:

(a) yes

(b) no

(2) age of menarche: 13 / 14 / 15

(3) paramenia:

(a) yes which and when

(b) no

(4) contraception:

no / spiral / pill / three month injection / contraception by spouse

(5) contraceptive means:

(a) from: to: name of the contraceptive:

from: to: name of the contraceptive:

(please also ask your gynecologist)

(b) can not be answered

(6) hormonal treatment for early climaterics and other dysfunction

(a) yes from to: name of the preparation:

(please ask your gynecologist)

(b) no

(7) did you notice any connection between the receipts of the contraceptives or other hormon preparations and increase of neurofibromas in number and size

(a) yes: slightly medium significant

(b) no

(8) did you notice any connetion between other events (e.g., receipts of medizine, change at work, psychological stress) and behaviour of your neurofibromas?

(a) yes

(b) no
